# The evaluation of alveolar fractures of trauma patients in Iran

**DOI:** 10.1186/s12903-021-01863-y

**Published:** 2021-10-06

**Authors:** Farhad Ghorbani, Mohammad Khalili, Hanie Ahmadi

**Affiliations:** 1 Department of Oral and Maxillofacial Surgery, School of Dentistry, Shiraz University of Medical Sciences, Shiraz, Iran; 2 Student Research Committee, Shiraz University of Medical Sciences, Shiraz, Iran

**Keywords:** Alveolar, Fracture, Trauma, Maxilla, Mandible

## Abstract

**Background:**

Alveolar bone plays a vital role in mastication and supporting the teeth. The alveolar process is one of the most challenging regions of facial bone to reconstruct due to the deformity involves both hard and soft tissues. However, the etiology, gender, and age distribution vary between different regions, cultures, and countries. This study aims to investigate the prevalence of alveolar trauma in Shahid Rajaee Hospital, Shiraz, Iran, for three years.

**Methods:**

In a retrospective cross-sectional study, patients with alveolar fractures referred to Shahid Rajaei Hospital in Shiraz were included in the study. Age, sex, site of alveolar fractures, and etiology factors of trauma explored. The collected data was analyzed by SPSS software. Mean $\pm$ SD calculated for the inferential statistics, and the data compared using Chi-square and Exact Fisher. A *p*-value of < 0.05 was considered statistically significant with a 95% reliability.

**Results:**

A total of 165 patients had alveolar fractures in this study. We found that the most common cause of alveolar fracture was road accidents (32.3%) and the lowest reason was violence (9%). Most people with alveolar trauma were male and in the 21–30 years. The prevalence of mandibular and maxillary alveolar fractures was 17.61 and 17.01%, respectively, with the most anterior area of injury.

**Conclusion:**

Alveolar trauma is one of the most common injuries among trauma patients. Early diagnosis and treatment plans are necessary to reduce the complications of facial trauma. Early training for a young adult is essential to prevent the severity of trauma.

## Background

Trauma is any damage to vital tissues caused by physical, chemical, or biological factors, and it can occur exclusively or combined with other injuries [1]. The etiology of trauma is different from one country to another still, road accidents, interpersonal violence, falling, and accident during the sport are the most common etiological factors of maxillofacial trauma [2]. Age, sex, and alcohol consumption are highly associated with injuries and severity of them [3].

The oral cavity accounts for only 5 percent of the total body, and about 1 percent of all body trauma confined to this region [4]. Facial bone and skin are more prone to trauma due to their anterior position, and the mandible is the most fractured bone associated with dental and soft tissue injuries [5, 6]. Craniofacial trauma expected at all ages, but it is less common in children and also, injuries are less severe in young children than in older ones [3, 7].

Maxillofacial injuries can occur as an isolated injury or may accompanied by multiple injuries in other parts of the body [8]. A high percentage of trauma patients suffer from alveolar fractures, including 32% of dental injuries, 27% of soft tissue injuries, and 1% of jaw bone fractures [9]. facial fractures include midface fractures—Le fort I, II, III, alveolar fractures in the maxilla and mandible, and mandible fractures including body, angle symphysis, para symphysis, and condyle [10].

Alveolar bone plays an important role in mastication and supporting the teeth. Alveolar fractures can categorized into the area surrounding a single tooth or regional type [11]. Loss of alveolar bone mostly occurs after severe traumatic events such as sports injury, violence, or accident. The alveolar process is one of the most challenging regions of facial bone to reconstruct due to the deformity involves both hard tissues and mucosa in mobile and moist conditions [12]. Alveolar fractures involve with displacement or fracture of teeth, the fracturing of bone, and injuring of soft tissue including abrasions, lacerations, and contusions [13, 14]. A radiograph should be taken when the signs of alveolar fractures are detected, and the gold standard for assesing facial injuries is Computed Tomography (CT) imaging [15]. There are some treatment choices in alveolar fractures, but the treatment chosen is depending on the patient's conditions; however, in most cases, open reduction with the internal fixation is preferred, and the few cases managed by closed reduction [16]. Studies recommended wire fixation for 4 to 6 weeks after alveolar fractures [17].

In Iran, the number of deaths caused by trauma increase due the urbanization and industrialization [18]. Basal bones of the mandible and maxilla reconstructed correctly, but rehabilitation of alveolar bone should considered for fixed and removable prosthetics, including, costly interventions such as bone grafting. Epidemiological study of alveolar trauma provides valuable information about the problems that occurred and successful management. Injuries of the jaws, including alveolar fractures, present in a small number of trauma patients, but the treatment of such injuries are too difficult to reconstruct. This study aims to investigate the prevalence of alveolar fracture in Shahid Rajaee Hospital, Shiraz, Iran, for three years.

## Methods

### Population and design

The Oral and Maxillofacial Surgery Center at Rajaee Hospital, Shiraz University of Medical Sciences, Shiraz, Iran, provides maxillofacial trauma treatment for many people in the Fars province.

This retrospective cross-sectional matched-control study done on all patients who had been referred to Shahid Rajaei Hospital, Trauma Center, between, 2016, to 2020. In this study, all individuals of different ages were examined, and there was no age limit.

Written informed consent was obtained from the participants and their anonymity was guaranteed. The study protocol was approved by the institutional research board (IRB) and the medical ethics committee of the Shiraz University of Medical Sciences by the IRB number1399.081REC.AL IR. SUMS.DENT.

All information was collected by reviewing the files in the archives during the mentioned years. The list of patients was prepared from the Hospital Information System (HIS), archived files, and CT images were assessed.

Inclusion criteria included all trauma patients of any age and sex who had referred to Shahid Rajaei Trauma Centers in Shiraz, completed the medical records, and consented to participate in the study. The exclusion criteria were prior maxillofacial surgeries, and inadequate imaging, and medical records of treatment or follow-up visits.

In the CT scan of the patient, various fractures, including fractures of different areas of the face, jaw, and alveolar process were recorded.

Age, sex, the etiological factors of trauma, and the site of the alveolar fractures were evaluated. The total number of patients with alveolar fractures and the anatomical location of the fractured jaw (anterior, posterior, and anterior–posterior) were measured.

The cause of trauma was divided into five main categories (i) car accident; (ii) motorcycles accident; (iii) violence; (iv) falling; and (v) others, which included domestic accidents, suicide attempts, pathological fractures, occupational accidents, accidents with animals, an accident during sports, fractures caused by teeth extraction and unknown etiology.

### Data gathering and analysis

Data were analyzed using the Statistical Package for Social Sciences (SPSS, version. 22.0, SPSS Inc., Chicago, IL, USA).

Mean $\pm$ SD was calculated for the inferential statistics and the data were compared using Chi-square and Exact Fisher. A p-value of < 0.05 was considered statistically significant with a 95% reliability.

## Results

In the three years of this study, 477 patients were diagnosed with maxillofacial trauma, and 375 (78.6%) of whom were male, 102 (21.3%) were female and the difference was statistically significant (chi-squared test, *p *value < 0.005). Among them, 165 (34.5%) had alveolar trauma. The mean age of male patients was 08/10 ± 58/26 years, female 13/11 ± 03/30 years, and this difference was statistically significant (p-value = 0.006). facial trauma was more frequent in the age group of 21–30 years (n = 190, 39.8%) followed by below 20 years (n = 145, 30.3%), and 31–40 years (n = 92, 19,3%). The most common cause of alveolar fracture was due to car accidents (n = 154, 32.3%) and the lowest cause was related to violence (n = 43, 9%) (Table 1).

**Table 1:** Demographic data and etiology of maxillofacial trauma

Variables	Number	Percentage
Gender		
Male	375	78.6
Female	102	21.3
Age		
Below 20	145	30.3
21–30	190	39.8
31–40	92	19.3
41–50	32	6.70
Over 50	18	3.77
Etiology		
Motorcycle accident	146	30.6
Car accident	154	32.3
Falling down	58	12.2
Violence	43	9
Other	73	15.3

The prevalence of maxillary alveolar fracture was 81 (17.01%). furthermore, the most commonly affected location was anterior area (n = 42, 8.8%) followed by posterior area (n = 27, 5.5%), and anterior–posterior area (n = 12, 2.5%). Our study showed that in the age of 21–30 years the prevalence of maxillary alveolar fractures was more common in the anterior region (n = 12, 14.8%). However, a negative correlation and insignificant association were found between maxillary alveolar fractures and age groups (r = − 0.042, *p *value = 0.711) (Table 2). Our results show that anterior maxillary alveolar fracture commonly accompanied by injuries in the nasal-zygoma-lefort of facial bone (n = 8, 14.5%), but a negative correlation and insignificant association were found between them (r = − 0.188, *p *value = 0.17). prevalence of maxillary alveolar fracture based on mandibular fractures showed that anterior maxillary alveolar fracture was frequently accompanied with injuries in the body of the mandible (n = 6, 26.1%) and but a negative correlation and insignificant association were found between them (r = − 0.347, *p *value = 0.105).

**Table 2:** The prevalence of alveolar fractures according to age

	Age										Spearman correlation	*p*-value
	Below 20		21–30		31–40		41–50		Over 50			
	Number	Percentage	Number	Percentage	Number	Percentage	Number	Percentage	Number	Percentage		
Maxilla												
Anterior site	17	20.9	12	14.8	8	9.8	1	1.2	3	3.7	−0.085	0.44
Posterior site	3	3.7	11	13.5	9	11.1	3	3.7	1	1.2		
Anterior and posterior site	2	2.4	2	2.4	2	2.4	4	4.9	3	3.7		
Mandible												
Anterior site	17	20.2	13	15.4	7	8.3	1	1.1	3	3.5	−0.042	0.711
Posterior site	4	4.7	13	15.4	8	9.5	4	4.7	2	2.3		
Anterior and posterior site	1	1.1	3	3.5	3	3.5	2	2.3	3	3.5		

Table 3 shows that the prevalence of maxillary alveolar fractures was more common in the male with anterior trauma (n = 36, 44.4%). Besides, there was a negative correlation between maxillary alveolar fractures and gender (r = − 0.40) though, it was not significant (P-value = 0.723).

**Table 3:** The prevalence of alveolar fractures according to gender

	Male		Female		Spearman correlation	*p*-value
	Number	Percentage	Number	Percentage		
Maxilla						
Anterior site	36	44.4	6	7.4	−0.40	0.723
Posterior site	20	24.7	7	8.6		
Anterior and posterior site	7	8.6	5	6.2		
Mandible						
Anterior site	34	40.5	7	8.3	−0.085	0.44
Posterior site	26	31	6	7.1		
Anterior and posterior site	29	35.4	4	4.8		

Generally, patients with anterior maxillary alveolar trauma caused by motorcycle vehicle accidents had the highest number (n = 18, 22.2%). However, a negative correlation and insignificant association were found between etiology and type of maxillary alveolar fractures (r = 0.048 and *p*-value = 0.67) (Table 4).

**Table 4:** The prevalence of alveolar fractures according to etiologies

	Etiology										Spearman correlation	*P *value
	Motorcycle accident		Car accident		Falling down		Violence		Other			
	Number	Percentage	Number	Percentage	Number	Percentage	Number	Percentage	Number	Percentage		
Maxilla												
Anterior site	18	22.2	8	9.9	1	1.2	7	8.6	8	9.9	0.048	0.67
Posterior site	8	9.9	7	8.6	1	1.2	5	6.2	6	7.4		
Anterior and posterior site	0	0	6	7.4	1	1.2	2	2.5	3	3.7		
Mandible												
Anterior site	19	22.6	10	11.9	2	2.4	3	3.6	7	8.3	−0.087	0.43
Posterior site	7	8.3	11	13.1	4	4.7	3	3.6	7	8.3		
Anterior and posterior site	27	32.1	27	32.1	7	8.3	7	8.3	16	19		

A total of 84 patients (17.61%) had mandibular alveolar fractures. The most common fracture site was anterior region (n = 41, 8.6%) followed by posterior region (n = 32, 6.7%), and anterior–posterior (n = 11, 2.3%). It was found that in the age of 21–30 years the prevalence of mandibular alveolar fractures in the anterior and posterior site was even (n = 13, 15.4%). Besides, there was a negative correlation between mandibular alveolar fractures and age groups (r = − 0.085) however, it was not significant (*P* value = 0.44) (Table 2). Our results show that anterior mandibular alveolar fracture was commonly accompanied by injuries in the nasal-zygoma-lefort of facial bone (n = 6, 14.6%) still a negative correlation and insignificant association was found between them (r = − 0.160, *p*-value = 0.31). Prevalence of mandibular alveolar fracture based on maxillary fractures showed that anterior mandibular alveolar fracture frequently accompanied with injuries in the symphysis of the mandible (n = 16, 33.3%) and but a negative correlation and insignificant association found between them (r = − 0.181, *p*-value = 0.219).

Table 3 shows that the prevalence of mandibular alveolar fractures was more common in the male with trauma in the anterior site (n = 34, 40.5%). Additionally, there was a negative correlation between mandibular alveolar fractures and gender (r = − 0.085) though, it was not significant (*P*-value = 0.44).

Overall, patients with anterior mandibular alveolar trauma caused by motorcycle vehicle accidents had the highest number (n = 19, 22.6%). However, a negative correlation and insignificant association were found between etiology and type of mandibular alveolar fractures (r = − 0.087 and *p*-value = 0.43) (Table 4).

## Discussion

Alveolar fractures are frequent among people due to enhancements in technology and the increase in the use of motor vehicle equipment. Early diagnosis and appropriate management play an essential role in the success of the treatment and can prevent tooth or alveolar loss.

Although the leading causes of facial trauma are road crashes and violence, the relationship between these causes varies with culture, socioeconomic status, and geographic region [19]. Our study revealed that the most common cause of alveolar fractures was due to the car accident and the most minor common cause was violence. Many studies have reported road accidents as the leading cause of maxillofacial trauma [20–23]. Hongwei Wang et al. found that the most common etiologies of facial fractures were motor vehicle accidents (39.0%), followed by high falling down (26.0%), and low falling down (20.8%) [24]. One of the Iranian studies reported that the most and least common reasons for maxillofacial fracture were road accidents and falling, respectively [25].

On the other hand, most other studies revealed that falling and violence were the main causes of facial injuries [26, 27]. This strongly supports the fact that falls and accidents would be more associated with alveolar fractures in adults. This difference in the main etiology of facial fractures varies from one country to another. It can influence by age, culture, social and economic factors [28]. In children, falling, sports injuries, and bicycle injuries can be the main cause of alveolar fractures. But at an older age, violence, accidents, falling, and work-related injuries can be the main cause of facial trauma [29].

The ratio of men to women was 78.6% to 21.2.% in our study. This is consistent with previous studies indicated that maxillofacial trauma is more frequent in men [30–33]. However, studies reported that women have a higher risk of maxillofacial fractures with dental trauma [34]. This report suggested that men are prone to alveolar injuries due to the more likely to be present in the community, face violence, and have more car-related occupations. A Swedish investigation found a positive relationship between violence and drinking among youth. Alcohol consumption among younger men can connect to violence; therefore, it could be a reason for the higher incidence of alveolar fractures [35].

In most studies, the highest incidence of alveolar fractures found in youth [36, 37]. In our study, the means age of men and women was 26.58 ± 10.08 and 30.03 ± 11resepctively. Our results show that most people with alveolar fractures were between 21 and 30 years old. The gender and age distribution are very similar to previous studies, indicated that young men are the vast majority of patients with alveolar fractures [38, 39]. Also, with the increase in age, the prevalence of alveolar injuries is going to be decreased [36, 37].

In this research, a total number of 165 (34.5%) had alveolar fractures for three years. Studies showed that trauma accounts for 5.5% of bone alveolar fractures, and 47–58% for soft tissue injuries [40, 41]. Another study of fractures in pediatric patients during 15 years reported that mandibular fractures were the most common (56%), then alveolar bone fractures (31%) [42]. At the Osaka University Dental Hospital, the most common form of maxillofacial injury was mandibular fractures (56%), followed by alveolar fractures (31%) [43]. An alveolar bone fracture can involve both dentate and edentulous arches [11]. In most cases, alveolar fractures and dental trauma occur simultaneously and the treatment of alveolar bone influences the treatment of teeth [34]. Alveolar fractures accompanied by dental fractures are more complicated in children than adults [34].

In our investigation, the prevalence of mandibular and maxillary alveolar fracture was 17.61% and 11.1%, respectively and most of their trauma was anterior site. This is compatible with other studies that reported the anterior maxilla and mandible are common sites due to the location and vulnerability of these regions [44]. Incisor over-jet, proclination, and incompetent lips of the upper arch and the low bone density of the anterior mandible make the anterior site of both jaws more prone to alveolar fracture [11, 45].

In most of the patients, alveolar fractures occurred with other facial bone fractures. Ruslin et al. stated that fractures are more common in the lower third of the face [34]. However, some researchers found that the upper two-thirds of the face are more susceptible to fractures [46, 47]. Previous studies found that the most common sites of fracture are mandibular bone, followed by zygomatic and orbital bone [48]. Furthermore, the mandibular fractures tend to happen more often in, condyle and body respectively [48].

Alveolar trauma needs special attention in geriatric patients due to some physiological changes in the elderly population such as bone atrophy, inadequate blood supply, and postponement in tissue repair [49]. In the above 70 years old individuals, facial trauma is progressively more frequent in women than in men due to the longer life of women [50]. The previous investigation stated that falls are the most frequent cause of maxillofacial trauma in the geriatric population [50]. The surgical management of alveolar fractures in elderly patients should be focused not only on the maxillofacial site but also on the other parts of the body that may need complex multidisciplinary management [49].

The results of our study may have been affected by some limitations. One of the most critical weaknesses of our study is that all cases in this research were patients that referred to the Rajaee hospital, and the sample might not include the whole population. Another restriction is that all of our participants were Iranian, while patients from different countries should be examined to find etiological factors of facial trauma. The differences between age, gender, and etiologies should analyzed to see the differences. The most common day of the week or time of the year that patients suffer from maxillofacial trauma could be evaluated to see an association between facial trauma and particular time like holidays. A rise in the number of participants of both genders and different age range recommended to evaluate alveolar fractures.

## Conclusion

This study showed that alveolar trauma is one of the most common injuries among trauma patients. The most crucial incidence factor of these injuries is road accidents, and the majority of patients are young men; however, the prevalence of alveolar fractures is going to decrease with age. Early diagnosis and treatment plans are necessary to reduce the complications after facial trauma. Early training for young adults is vital to prevent the severity of trauma.

## Data Availability

The datasets generated during and/or analysed for the current study are available from the corresponding author on reasonable request.

## Abbreviations

CT: Computed Tomography
HIS: Hospital Information System
SPSS: Statistical Package for Social Sciences
